# Complete plastid genome of *Litsea honghoensis* Liou 1933, an endangered evergreen species in China

**DOI:** 10.1080/23802359.2022.2122884

**Published:** 2022-09-23

**Authors:** Lihong Han, Jian Cai, Huanhuan Chen, Chao Liu

**Affiliations:** College of Biological Resource and Food Engineering, Yunnan Engineering Research Center of Fruit Wine, Qujing Normal University, Qujing, China

**Keywords:** *Litsea*, chloroplast, phylogeny

## Abstract

*Litsea honghoensis* Liou is an endangered tree endemic to south China. In this study, the first complete plastid genome of *L. honghoensis* was presented, which had a length of 152,605 base pairs (bp) with a GC content of 39.20%. The genome consisted of a large single-copy (LSC) region of 93,560 bp, a small single-copy (SSC) region of 18,905 bp, and two inverted repeat regions (IRa and IRb) of 20,070 bp. There were 125 genes in the plastid genome, including 81 protein-coding genes, 36 transfer RNA (tRNA) genes, and eight ribosomal RNA (rRNA) genes. Phylogenomic analysis based on 52 complete plastomes of Laureae in the family Lauraceae supports the close relationships among *L. honghoensis*, *Lindera communis*, *Lindera nacusua*, *Lindera angustifolia*, and *Lindera glauca*.

*Litsea honghoensis* Liou 1933 as an evergreen species of the family Lauraceae that is naturally distributed in the valley forests in the southeast and south of Yunnan, China (http://foc.iplant.cn/). It belongs to the genus *Litsea* Lamarck distributed mainly in tropical or subtropical Asia and North or South America (http://www.plantsoftheworldonline.org/). It is a key protected rare and vulnerable (VU) species in China (Qin et al. 2017), because of habitat degradation or loss, direct excavation or cutting, intrinsic factors and interspecific influence. However, there has been no genomic study on the *L. honghoensis*, which greatly limit the protection and utilization of *L. honghoensis*. *Litsea* is often confused with genus *Lindera* and *Laurus* in the core Lauraceae (Zhao et al. 2018; Tian et al. 2019; Liu et al. 2020; Song et al. 2020). In order to understand the relationships of *L. honghoensis* and other Laureae species, we assembled and annotated the plastid genome of *L. honghoensis* as a resource for evolution and protection research.

The leaf samples of *L. honghoensis* were collected from Kunming Arboretum (Kunming, China; Long. 102.740333 E, Lat. 25.134722 N, 1950 m). The voucher specimens (accession number: XTBG-BRG-SY35037) were deposited at the Herbarium of Xishuangbanna Tropical Botanical Garden. Total genomic DNA was extracted using the CTAB method (Doyle and Dickson 1987). A paired-end library of 350 bp was constructed. Approximately, 1.8 Gb of raw data of 150-bp-long paired-end reads were generated using the Illumina NovaSeq platform. The plastid genome of *L. honghoensis* was assembled and annotated using Geneious 4.8 and GeSeq (Tillich et al. 2017) with *Lindera glauca* (MG581443) served as the reference. The complete plastid genome of *L. honghoensis* was submitted to NCBI (accession number OL362095).

The whole plastid genome of *L. honghoensis* was 152,605 base pairs (bp) in length. The plastid genome exhibited a typical quadripartite structure with a large single-copy (LSC) region of 93,560 bp, a small single-copy (SSC) region of 18,905 bp, and two inverted repeat regions (IRs) of 20,070 bp. The plastid genome contained 125 genes, including 81 protein-coding genes, 36 transfer RNA (tRNA) genes, and eight ribosomal RNA (rRNA) genes. The G + C content of the whole plastid genome was 39.20%, and those of LSC region, SSC region, and IR region were 38.02%, 33.93%, and 44.42%, respectively.

To further investigate the phylogenetic position of *L. honghoensis*, 52 complete plastid genome sequences in Laureae were aligned using MAFFT v. 7.490 (Katoh et al. 2019). Maximum-likelihood (ML) phylogenetic analyses were performed based on TIM + F+R2 model in the IQ-TREE v. 2.1.1 with 1000 bootstrap replicates (Figure 1). The ML phylogenetic tree with 57–100% bootstrap values at each node showed that *Litsea* species was grouped into two clades, and that *L. glutinosa*, *L. magnifolia*, *L. acutivena*, *L. honghoensis*, *L. pungens*, and *L. tsinlingensis* were located in the same clade, while *L. dilleniifolia*, *L. szemaois*, *L. coreana*, *L. coreana* var. *sinensis*, *L. elongata*, *L. garrettii*, *L. monopetala*, *L. mollis*, *L. cubeba*, *L. japonica*, *L. panamonja*, and *L. pierrei* in another clade. *L. honghoensis* is closed related to *Lindera communis*, *Lindera nacusua*, *Lindera angustifolia*, and *Lindera glauca* with 100% bootstrap value. The *Litsea* is polyphyletic and closely related to the genera *Lindera* and *Laurus*, which is consistent with previous studies (Xiao et al. 2020; Zhang et al. 2021; Liu et al. 2022).

**Figure 1.**
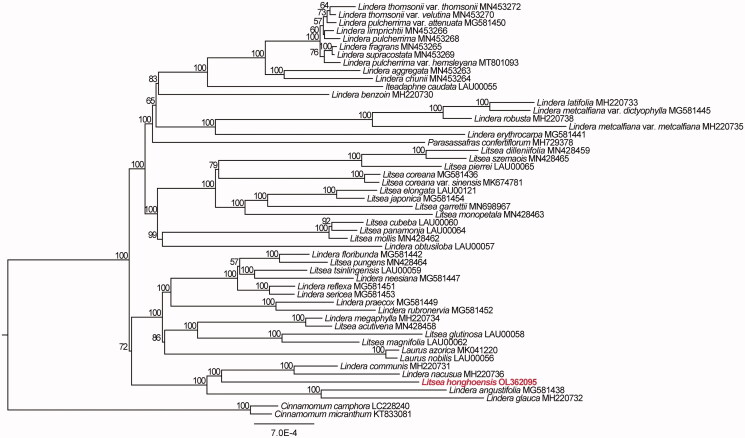
The maximum-likelihood phylogenetic tree constructed with plastid genomes of *L. honghoensis* and other 51 taxa.

## Data Availability

The genome sequence data that support the findings of this study are openly available in GenBank of NCBI at (https://www.ncbi.nlm.nih.gov/) under the accession no. OL362095. The associated BioProject, SRA, and Bio-Sample numbers are PRJNA778331, SRR16832243, and SAMN22942525, respectively.
